# Creation and Transfer of Coherence via Technique of Stimulated Raman Adiabatic Passage in Triple Quantum Dots

**DOI:** 10.1186/s11671-016-1433-6

**Published:** 2016-04-23

**Authors:** Si-Cong Tian, Ren-Gang Wan, Chun-Liang Wang, Shi-Li Shu, Li-Jie Wang, Chun-Zhu Tong

**Affiliations:** State Key Laboratory of Luminescence and Applications, Changchun Institute of Optics, Fine Mechanics and Physics, Chinese Academy of Sciences, Changchun, 130033 China; School of Physics and Information Technology, Shaanxi Normal University, Xi’an, 710062 China; Centre for Advanced Optoelectronic Functional Materials Research and Key Laboratory for UV Light-Emitting Materials and Technology of Ministry of Education, Northeast Normal University, Changchun, 130024 China

**Keywords:** Triple quantum dots, Creation and transfer of coherence, Stimulated Raman adiabatic passage

## Abstract

We propose a scheme for creation and transfer of coherence among ground state and indirect exciton states of triple quantum dots via the technique of stimulated Raman adiabatic passage. Compared with the traditional stimulated Raman adiabatic passage, the Stokes laser pulse is replaced by the tunneling pulse, which can be controlled by the externally applied voltages. By varying the amplitudes and sequences of the pump and tunneling pulses, a complete coherence transfer or an equal coherence distribution among multiple states can be obtained. The investigations can provide further insight for the experimental development of controllable coherence transfer in semiconductor structure and may have potential applications in quantum information processing.

## Background

Atomic coherence has attracted considerable interest in recent years because atomic coherence is essential for many effects, such as electromagnetically induced transparency (EIT) [1–3], laser without inversion [4–6], coherent population transfer [7–10], and subluminal and superluminal light propagation [11, 12]. The technique of stimulated Raman adiabatic passage (STIRAP) can be used for coherent controlling of an atomic system to a particular state, both in a Λ-type three-level system [13, 14] and in a multiple-level system [15–19]. And by fractional STIRAP (F-STIRAP), creation of atomic coherence can be obtained [20]. Besides, in a Λ-type system where the final state has twofold states, creation of atomic coherence by STIRAP is also possible because of the double dark states induced by the control laser [21].

On the other hand, quantum dots (QDs) have three-dimensional confinement of carriers, which makes the holes and electrons in QDs only occupy the discrete-energy states. Compared with atoms, QDs have larger electric-dipole moments and higher nonlinear optical coefficients. Besides, the other advantages of QDs over atoms are their flexible designed energy scales and physical features, proper selection of the materials and the sizes, and customized design and ease of integration. Thus, QDs are widely used to perform atomic coherence experimental and theoretical investigations in solid-state structures. For instance, coherent manipulation population [22–25] and other coherent phenomena [26–28] in QDs have been reported.

The next natural step is to couple two closely spaced QDs together. By the self-assembled dot growth method, double quantum dots (DQDs) can be fabricated [29]. In DQDs, the tunneling between the inter dots can be controlled not only by the composition but also by the externally applied voltages; thus, DQDs are an ideal system for experimental and theoretical investigations, where the interactions between light and matter can be fully controlled and coherence characteristics can be probed by electrical and optical methods. Therefore, many studies concentrate on generating and employing the coherence in DQDs [30–40]. Based on DQDs, triple quantum dots (TQDs) have been fabricated in many processes [41–44]. The electron and hole confinement, as well as the intermediate band of such quantum dot molecules, have been studied [45, 46]. And TQDs can bring in multilevel structure and extra controlling parameters which cannot be found in DQDs [47–49].

We note that the coherent population transfer can be realized in a three-level QD system with more than one dot [50–52]. But to our knowledge, there is no investigation on transferring and manipulating of coherence. So in this paper, we propose a scheme for controlling coherence transfer among ground state and indirect exciton states of TQDs via the technique of STIRAP. Compared with the traditional STIRAP, the Stokes laser pulse is replaced by the tunneling pulse, which can be controlled by the externally applied voltages. We show that a complete coherence transfer or an equal coherence distribution among multiple states can be obtained via the pump and tunneling pulses with different amplitudes and sequences. Our investigations can provide further insight for the experimental development of controllable coherence transfer in semiconductor structure and may have potential applications in quantum information processing.

## Methods

**Fig. 1:**
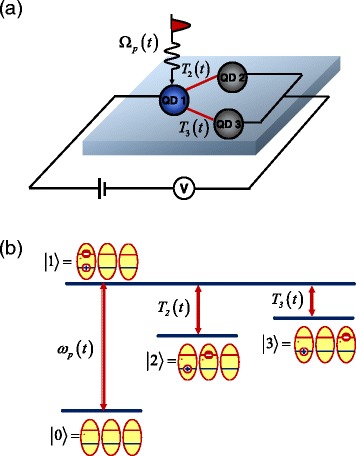
**a** The schematic of the setup of the TQDs. The pump pulse transmits QD 1. **b** The schematic of the level configuration of the TQDs

The TQD system consists of three QDs, which have different band structures and are arranged triangularly, as shown in Fig. 1a. In such system, the tunneling barrier depends on the gate electrode between the three QDs. When the gate voltage is not applied, the conduction-band electron energy levels are out of resonance; therefore, the electron tunneling between the neighbor QDs is quite weak. On the contrary, when the gate voltage is applied, the conduction-band electron energy levels are resonant; therefore, the electron tunneling between the neighbor QDs becomes very strong. The hole tunneling is neglected due to the off-resonance of the valence-band energy levels in the latter situation.

Under the resonant coupling of a pump laser field with QD1, an electron is excited in QD1. Then with the tunneling, the electron can be transferred to QD2 and QD3. Thus, the TQD structure can be treated as a four-level tripod system (Fig. 1b): the ground state |0〉, where there is no excitations in any QD, the direct exciton state |1〉, where the electron and hole are both in the first QD, the indirect exciton state |2〉, where the electron is in the second dot and the hole remains in the first dot, and the indirect exciton state |3〉, where the electron is in the third dot and the hole remains in the first dot.

At any time *t*, the state vector can be written as

1 $$ \Big|\varPsi (t)\rangle \kern0.5em =\kern0.5em {a}_0(t)\Big|0\rangle \kern0.5em +\kern0.5em {a}_1(t)\Big|1\rangle \kern0.5em +\kern0.5em {a}_2(t)\Big|2\rangle \kern0.5em +\kern0.5em {a}_3(t)\Big|3\rangle . $$

The time evolution of the probability amplitude *A*(*t*) = [*a*_0_(*t*), *a*_1_(*t*), *a*_2_(*t*), *a*_3_(*t*)]^*T*^ of the four states is described by the Schrödinger equation

2 $$ \frac{d}{dt}A(t)\kern0.5em =\kern0.5em -\kern0.5em \frac{i}{\hslash }H(t)A(t)\kern0.5em -\kern0.5em \varLambda A(t), $$

where *H*(*t*) is the Hamiltonian of TQDs and *Λ* is the dissipative process containing spontaneous decay process and the pure dephasing. In the rotating-wave approximation, the expression of *H*(*t*) under the coupling of the pump and tunneling pulses can be written as

3 $$ H(t)=\hslash \left(\begin{array}{cccc}\hfill 0\hfill & \hfill {\varOmega}_p(t)\hfill & \hfill 0\hfill & \hfill 0\hfill \\ {}\hfill {\varOmega}_p(t)\hfill & \hfill -{\delta}_p\hfill & \hfill {T}_2(t)\hfill & \hfill {T}_3(t)\hfill \\ {}\hfill 0\hfill & \hfill {T}_2(t)\hfill & \hfill -\left({\delta}_p-{\omega}_{12}\right)\hfill & \hfill 0\hfill \\ {}\hfill 0\hfill & \hfill {T}_3(t)\hfill & \hfill 0\hfill & \hfill -\left({\delta}_p-{\omega}_{13}\right)\hfill \end{array}\right). $$

Here, *Ω*_*p*_(*t*) is the Rabi frequency of the pump pulse, and *T*_2_(*t*) and *T*_3_(*t*) are the tunneling pulses, which can be controlled by varying the bias voltage. In our calculations, *Ω*_*p*_, *T*_2_, and *T*_3_ denote the peak value of the pump pulse and two tunneling pulses, and all the pulses have the same pulse duration *T*. The energy splitting of the direct exciton state |1〉 and ground state |0〉 is *ω*_10_, and the energy splitting of the direct exciton state |1〉 and indirect exciton states |2〉 and |3〉 are *ω*_12_ and *ω*_13_, respectively. *δ*_*p*_ = *ω*_10_ − *ω*_*p*_ denotes the pump detuning (*ω*_*p*_ is the frequency of the pump pulse). And in TQDs, the energy splitting depends on the effective confinement potential and are much smaller than *ω*_10_.

Substituting Eq. (1) and Eq. (3) into Eq. (2), we can obtain the following dynamical equations for atomic probability amplitudes in the interaction picture:

4a $$ i{\overset{.}{a}}_0=-{\varOmega}_p{a}_1, $$

4b $$ i{\overset{.}{a}}_1=-{\varOmega}_p{a}_0-{T}_2{a}_2-{T}_3{a}_3+\left({\delta}_p-i{\gamma}_1\right){a}_1, $$

4c $$ i{\overset{.}{a}}_2=-{T}_2{a}_1+\left({\delta}_p-{\omega}_{12}-i{\gamma}_2\right){a}_2, $$

4d $$ i{\overset{.}{a}}_3=-{T}_3{a}_1+\left({\delta}_p-{\omega_1}_3-i{\gamma}_3\right){a}_3, $$

Here, ${\gamma}_i\kern0.5em =\kern0.5em \frac{1}{2}{\varGamma}_{i0}+{\gamma}_{i0}^d\ \left(i\kern0.5em =\kern0.5em 1\hbox{--} 3\right)$ is the typical effective decay rate, with *Γ*_*i*0_ being the radiative decay rate of populations from |*i*〉 → |0〉 and ${\gamma}_{i0}^d$ being the pure dephasing rates.

The time evolutions of population and the coherence dynamics can be calculated by the density matrix element |*ρ*_*ij*_| = |*a*_i_*a_*j*_|. If *i* = *j*, |*ρ*_*ij*_| represents the time evolutions of population *P*_*i*_, while if *i* ≠ *j*, |*ρ*_*ij*_| represents the coherence dynamics.

In our calculations, the realistic values of TQD parameters are *ℏT*_2,3_ ~ 1–10 meV, *ℏγ*_1_ ~ 0.002–0.01 meV, and *γ*_2_ = *γ*_3_ = 10^− 3^*γ*_1_ [47]. And for simplicity, *δ*_*p*_, *ω*_12_, and *ω*_13_ are set to 0. With these parameters, the adiabatic condition can be fully satisfied. And in all the cases, the initial population is assumed to be in state |0〉, that is *a*_0_(−∞) = 1, *a*_1,2,3_(−∞) = 0.

## Results and Discussion

**Fig. 2:**
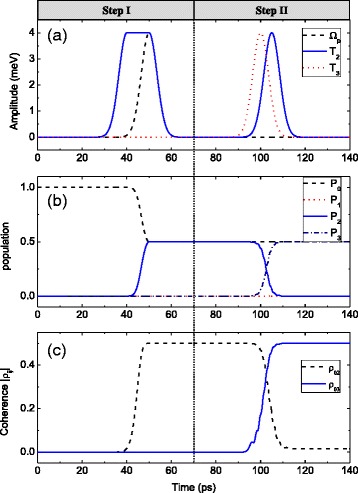
**a** The pump and the tunneling pulses. **b** The time evolutions of population *P*_*i*_ = |*a*_*i*_|^2^ (*i* = 0–3). **c** The coherence dynamics |*ρ*_*ij*_|. In step I, the peak values of *ℏΩ*_*p*_ and *ℏT*_2_ are both 4 meV. In step II, the peak values of *ℏT*_2_ and *ℏT*_3_ are both 4 meV. Other parameters are *δ*_*p*_ = *ω*_12_ = *ω*_13_ = 0, *ℏγ*_1_ = 0.01 meV, and *γ*_2_ = *γ*_3_ = *γ*_4_ = 10^− 3^ *γ*_1_

Our first task is to achieve coherence transfer in TQDs, and we show the corresponding results in Fig. 2. In step I, we prepare the coherence between states |0〉 and |2〉 by a F-STIRAP among states |0〉, |1〉, and |2〉. With the tunneling pulse *T*_2_(*t*) and the pump pulse *Ω*_*p*_(*t*), the system state vector in step I goes to

5a $$ \left|{\varPsi}_I\right\rangle = \cos \theta \left|0\right\rangle - \sin \theta \left|2\right\rangle, $$

5b $$ \tan \theta =\frac{\varOmega_p(t)}{T_2(t)}. $$

Here, the mixing angle *θ* is similar to the conventional one defined in STIRAP of a *Λ* atomic system. From Eq. (5a), state |*Ψ*_*I*_〉 has no component of state |1〉, which indicates that it does not arouse the stimulated emission from state |1〉 to |0〉 in step I. Furthermore, from Eq. (5a), the coherence amplitude between states |0〉 and |2〉 can be calculated, which is

6 $$ \left|{\rho}_{02}\right|=\left| \cos \theta \sin \theta \right|. $$

Hence, by tuning the mixing angle *θ*, arbitrary intensity of coherence between states |0〉 and |2〉 can be obtained. If *T*_2_(*t*) precedes *Ω*_*p*_(*t*), and they have the same amplitude and are switched off simultaneously, as time progresses from 0 to ∞, the value of *Ω*_*p*_(*t*)/*T*_2_(*t*) rises from 0 to 1, and consequently, the mixing angle *θ* rises from 0 to *π*/4. As a result, the adiabatic state |*Ψ*_*I*_〉 starting in the bare state |0〉 will end in the coherent superposition state $\left|{\varPsi}_I\right\rangle =\left(\left|0\right\rangle -\left|2\right\rangle \right)/\sqrt{2}$. This means that the maximal coherence between states |0〉 and |2〉 is obtained.

We give numerical simulation to illustrate the time evolutions of the system in step I. The tunneling pulse *T*_2_(*t*) and the pump pulse *Ω*_*p*_(*t*) are plotted in the left column of Fig. 2a. Then, the time evolutions of the population *P*_*i*_ = |*a*_*i*_|^2^ (*i* = 0–3) and the coherence dynamics |*ρ*_02,03_| can be drawn in the left column of Fig. 2b, c, respectively. As the left column of Fig. 2b reveals, the population is distributed equally in two states |0〉 and |2〉 at the end of step I. And state |1〉 is empty in the whole process. It can be seen from the left column of Fig. 2c that |*ρ*_02_| arises from 0 to the maximum value 1/2 during step I. Because *T*_3_(*t*) is switched off, the population *P*_3_ and the coherence |*ρ*_03_| remain 0.

Now, we have the coherence between states |0〉 and |2〉. Then in step II, we will transfer this coherence to that between states |0〉 and |3〉 by a STIRAP process among states |1〉, |2〉, and |3〉. In this process, *Ω*_*p*_(*t*) is switched off and both tunneling pulses *T*_2_(*t*) and *T*_3_(*t*) are switched on. Because the probability amplitude of |0〉 is unchanged, and the probability amplitude of |2〉 is changed to the superposition states of |2〉 and |3〉, the system state vector goes to

7a $$ \left|{\varPsi}_{II}\right\rangle = \cos \theta \left|0\right\rangle - \sin \theta \left( \cos \phi \left|2\right\rangle - \sin \phi \left|3\right\rangle \right), $$

7b $$ \tan \phi =\frac{T_2(t)}{T_3(t)}. $$

Here, *ϕ* is the other mixing angle relative to two tunneling pulses in the STIRAP process. From Eq. (7a), the state |*Ψ*_*II*_〉 has no component of the state |1〉 either, so it does not arouse the stimulated emission from state |1〉 to state |0〉 in step II. Also from Eq. (7a), the amplitudes of the possible coherence can be calculated

8a $$ \left|{\rho}_{02}\right|=\left| \cos \theta \sin \theta \cos \phi \right|, $$

8b $$ \left|{\rho}_{03}\right|=\left| \cos \theta \sin \theta \sin \phi \right|, $$

8c $$ \left|{\rho}_{23}\right|=\left|{ \sin}^2\theta \cos \phi \sin \phi \right|. $$

Hence, by tuning the mixing angle, the coherence among states |0〉, |2〉, and |3〉 with arbitrary value can be obtained. If *T*_3_(*t*) precedes *T*_2_(*t*) with the same amplitude, and they overlap in the process, as time progresses from 0 to ∞, the value of *T*_2_(*t*)/*T*_3_(*t*) rises from 0 to ∞, and consequently, the mixing angle *ϕ* rises from 0 to *π*/2. Together with *θ* = *π*/4 (step I), the final adiabatic state |*Ψ*_*II*_〉 will end in the coherent superposition state $\left|{\varPsi}_{II}\right\rangle \kern0.5em =\kern0.5em \left(\left|0\right\rangle \kern0.5em +\kern0.5em \left|3\right\rangle \right)/\sqrt{2}$. This means that the maximal coherence between states |0〉 and |3〉 is obtained.

We give numerical simulation to illustrate the time evolutions of the system in step II. The tunneling pulses *T*_2_(*t*) and *T*_3_(*t*) are plotted in the right column of Fig. 2a. Then, the time evolutions of the population *P*_*i*_ = |*a*_*i*_|^2^ (*i* = 0–3) and the coherence dynamics |*ρ*_02,03_| are shown in the right column of Fig. 2b, c, respectively. As can be seen in the right column of Fig. 2b, the population in state |2〉 is completely transferred to state |3〉, while the population in state |0〉 is unchanged. In the whole process, state |1〉 keeps empty. Furthermore, the right column of Fig. 2c reveals that the coherence between states |0〉 and |2〉 is fully transferred to that between states |0〉 and |3〉, with the maximum value being 1/2 at the end of step II.

**Fig. 3:**
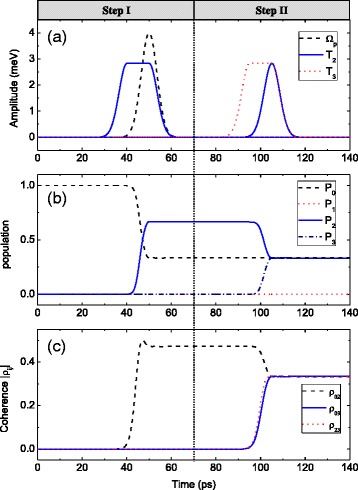
**a** The pump and the tunneling pulses. **b** The time evolutions of population *P*_*i*_ = |*a*_*i*_|^2^ (*i* = 0–3). **c** The coherence dynamics |*ρ*_*ij*_|. In step I, the peak values of *ℏΩ*_*p*_ and *ℏT*_2_ are 4 and 2.83 meV, respectively. In step II, the peak values of *ℏT*_2_ and *ℏT*_3_ are both 2.83 meV. Other parameters are the same as those in Fig. 2

Now, we focus our attention on how to control the coherence distribution in TQDs. We show the corresponding results in Fig. 3. Step I is to prepare the coherence between states |0〉 and |2〉. Here, we use the F-STIRAP among states |0〉, |1〉, and |2〉 and the pulse sequences are shown in the left column of Fig. 3a. Compared with Fig. 2a, the only difference is that the peak value of *Ω*_*p*_(*t*) and *T*_2_(*t*) is *Ω*_*p*_/*T*_2_ = 3/2 in Fig. 3a. As time progresses, the population transfers from state |0〉 to state |2〉, and *P*_0_ and *P*_2_ finally reach a stable value with the ratio of *P*_2_/*P*_0_ = 2 (left column of Fig. 3b). And the coherence between states |0〉 and |2〉 is also obtained, with the value of |*ρ*_02_| being a little smaller than the maximum value 1/2 (left column of Fig. 3c).

Step II is to distribute the obtained coherence to that between the desired states. In this process, we use another F-STIRAP process among states |1〉, |2〉, and |3〉 and show the pulse sequences in the right column of Fig. 3a. Both tunneling pulses have the same peak value and time back edge, and the peak value is 2/3 times that of the pump pulse in step I. As time goes, half of the population in state |2〉 is transferred to state |3〉, while the population in state |0〉 is unchanged. At last, the population is equally distributed in these three states |0〉, |2〉, and |3〉. And in the whole step II, state |1〉 is empty (right column of Fig. 3b). Besides, as can be seen in the right column of Fig. 3c, the value of |*ρ*_02_| decreases, while the value of |*ρ*_03_| and |*ρ*_23_| increases, and at last, all |*ρ*_02_|, |*ρ*_03_|, and |*ρ*_23_| reach a same stable value 1/3. This means that the coherence obtained in step I is successfully transferred to that between other states.

From Fig. 3, it can be concluded that using the technique of STIRAP, the maximum coherence among three states |0〉, |2〉, and |3〉 can be realized, with the value of coherence between arbitrary two states being 1/3. While in the usual multiple-level atomic system coupled by continuous-wave laser, the maximum value of coherence between two ground states is only 1/6 [53]. Besides, from Eqs. (6) and (8), the value of (|*ρ*_02_|^2^ + |*ρ*_03_|^2^)^1/2^ in step II is equal to that of |*ρ*_02_| in step I. In step I, the maximum value of |*ρ*_02_| is 1/2; therefore, arbitrary coherence distribution between states |0〉 and |2〉 and states |0〉 and |3〉 is only limited by (|*ρ*_02_|^2^ + |*ρ*_03_|^2^)^1/2^ ≤ 1/2.

**Fig. 4:**
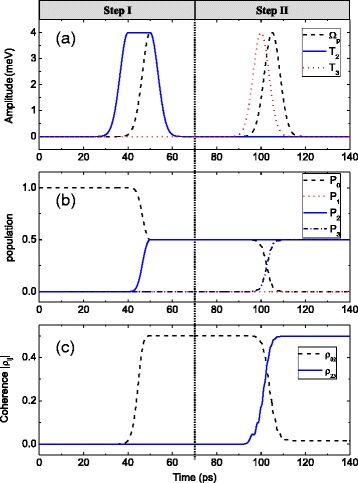
**a** The pump and the tunneling pulses. **b** The time evolutions of population *P*_*i*_ = |*a*_*i*_|^2^ (*i* = 0–3). **c** The coherence dynamics |*ρ*_*ij*_|. In step I, the peak values of *ℏΩ*_*p*_ and *ℏT*_2_ are both 4 meV. In step II, the peak values of *ℏΩ*_*p*_ and *ℏT*_3_ are both 4 meV. Other parameters are the same as those in Fig. 2

**Fig. 5:**
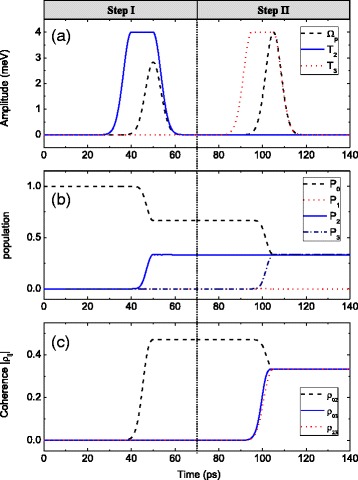
**a** The pump and the tunneling pulses. **b** The time evolutions of population *P*_*i*_ = |*a*_*i*_|^2^ (*i* = 0–3). **c** The coherence dynamics |*ρ*_*ij*_|. In step I, the peak values of *ℏΩ*_*p*_ and *ℏT*_2_ are 2.83 and 4 meV, respectively. In step II, the peak values of *ℏΩ*_*p*_ and *ℏT*_3_ are both 4 meV. Other parameters are the same as those in Fig. 2

The coherence transfer and coherence distribution can also been realized by other pulse sequences and amplitudes, and we show these results in Figs. 4 and 5, respectively. First, we show a complete coherence transfer. In the left column of Fig. 4a, step I prepares the coherence between states |0〉 and |2〉 by a F-STIRAP among states |0〉, |1〉, and |2〉, which is the same as step I of Fig. 2. Thus, half of the population is transferred from state |0〉 to state |2〉 (left column of Fig. 4b), and the maximum value of coherence between states |0〉 and |2〉 is obtained (left column of Fig. 4c). Then in step II, we will transfer the coherence to that between states |2〉 and |3〉. Different form Fig. 2, we use a STIRAP among states |0〉, |1〉, and |3〉, rather than states |1〉, |2〉, and |3〉, by applying the pump pulse *Ω*_*p*_(*t*) and tunneling pulse *T*_3_(*t*). During this process, the probability amplitude of |2〉 is unchanged, while the probability amplitude of |0〉 is changed to the superposition states of |0〉 and |3〉. In this case, the system state vector goes to

9a $$ \left|{\varPsi}_{II}\right\rangle = \cos \theta \left( \cos \phi \left|0\right\rangle - \sin \phi \left|3\right\rangle \right)- \sin \theta \left|2\right\rangle, $$

9b $$ \tan \theta =\frac{\varOmega_p(t)}{T_2(t)}, $$

9c $$ \tan \phi =\frac{\varOmega_p(t)}{T_3(t)}. $$

And as can be seen from Eq. (9a), |*Ψ*_*II*_〉 has no component of state |1〉. Here, *θ* and *ϕ* are the mixing angles. Then, the amplitudes of the possible coherence are

10b $$ \left|{\rho}_{02}\right|=\left| \cos \theta \sin \theta \cos \phi \right|, $$

10a $$ \left|{\rho}_{03}\right|=\left|{ \cos}^2\theta \sin \phi \cos \phi \right|, $$

10c $$ \left|{\rho}_{23}\right|=\left| \cos \theta \sin \theta \sin \phi \right|. $$

According to Eq. (10), it is possible to control the coherence by tuning the mixing angles. Thus, by using the pulse sequences in the right column of Fig. 4a, the population in state |0〉 is completely transferred to state |3〉 (right column of Fig. 4b), and the completed coherence transfer from states |0〉 and |2〉 to states |2〉 and |3〉 is realized (right column of Fig. 4c).

Next, according to Eq. (10), we present the coherence distribution and show the results in Fig. 5. Step I is to prepare the coherence between states |0〉 and |2〉, which can be realized by using the F-STIRAP among states |0〉, |1〉, and |2〉. The pulse sequences with the ratio of the peak value being *Ω*_*p*_/*T*_2_ = 2/3 are shown in the left column of Fig. 5a. As time progresses, some population transfers from state |0〉 to state |2〉 and the final ratio of the population is *P*_2_/*P*_0_ = 2 (left column of Fig. 5b). Meanwhile, the maximum coherence between states |0〉 and |2〉 is obtained (left column of Fig. 5c). In step II, another F-STIRAP process among states |0〉, |1〉, and |3〉 is used to distribute the obtained coherence. The pulse sequences are shown in the right column of Fig. 5a. As time goes, the population is equally distributed in three states |0〉, |2〉, and |3〉 (right column of Fig. 5b). At the same time, the coherence between states |0〉 and |2〉 is equally distributed to that between states |0〉 and |2〉, states |0〉 and |3〉, and states |2〉 and |3〉 (right column of Fig. 5c).

From Figs. 4 and 5, it can be concluded that it is possible to realize coherence transfer and coherence distribution by using other sequences and amplitudes of pump and tunneling pulses. From Eqs. (6) and (10), the value of (|*ρ*_02_|^2^ + |*ρ*_23_|^2^)^1/2^ in step II is equal to that of |*ρ*_02_| in step I. In step I, the maximum value of |*ρ*_02_| is 1/2; therefore, arbitrary coherence distribution between states |0〉 and |2〉 and states |2〉 and |3〉 is only limited by (|*ρ*_02_|^2^ + |*ρ*_23_|^2^)^1/2^ ≤ 1/2. So the limitation of coherence distribution may be different in the condition of the different pulse sequences.

## Conclusions

In this paper, we have theoretically demonstrated that it is possible to transfer and manipulate coherence among ground state and indirect exciton states of TQDs by the technique of STIRAP. The whole process can be separated into two steps; in the first step, the creation of coherence between the ground state and one indirect exciton state can be achieved by one process of F-STIRAP. Then in the second step, the complete transfer of coherence between the ground state and the other indirect exciton state can be obtained by the process of STIRAP, or the equal distribution of coherence among the ground state and two indirect exciton states can be obtained by the other process of F-STIRAP. These results can also be obtained by other pump and tunneling pulses with different amplitudes and sequences. Moreover, the value of equal coherence distribution among the multiple states by the technique of STIRAP can reach to 1/3, which is larger than that of using continuous-wave laser. And the only limitation of the coherence distribution is limited by the value of coherence |*ρ*_02_| in the first step. Our scheme allows controlling and manipulating coherence in a reliable and flexible way and may have essential applications in quantum information processing based on the atomic coherence effect, such as slow-light storage and quantum logical gates.
